# High-Performance Dye-Sensitized Solar Cells Based on Morphology-Controllable Synthesis of ZnO–ZnS Heterostructure Nanocone Photoanodes

**DOI:** 10.1371/journal.pone.0123433

**Published:** 2015-04-13

**Authors:** Jalal Rouhi, Mohamad Hafiz Mamat, C. H. Raymond Ooi, Shahrom Mahmud, Mohamad Rusop Mahmood

**Affiliations:** 1 Centre of Nanoscience and Nanotechnology (NANO-SciTech Centre), Institute of Science, Universiti Teknologi MARA, 40450 Shah Alam, Selangor, Malaysia; 2 NANO-ElecTronic Centre, Faculty of Electrical Engineering, Universiti Teknologi MARA, 40450 Shah Alam, Selangor, Malaysia; 3 Department of Physics, University of Malaya, 50603 Kuala Lumpur, Malaysia; 4 Nano-Optoelectronic Research (NOR) Lab, School of Physics, Universiti Sains Malaysia, 11800 Pulau Pinang, Pinang, Malaysia; Institute for Materials Science, GERMANY

## Abstract

High-density and well-aligned ZnO–ZnS core–shell nanocone arrays were synthesized on fluorine-doped tin oxide glass substrate using a facile and cost-effective two-step approach. In this synthetic process, the ZnO nanocones act as the template and provide Zn^2+^ ions for the ZnS shell formation. The photoluminescence spectrum indicates remarkably enhanced luminescence intensity and a small redshift in the UV region, which can be associated with the strain caused by the lattice mismatch between ZnO and ZnS. The obtained diffuse reflectance spectra show that the nanocone-based heterostructure reduces the light reflection in a broad spectral range and is much more effective than the bare ZnO nanocone and nanorod structures. Dye-sensitized solar cells based on the heterostructure ZnO–ZnS nanocones are assembled, and high conversion efficiency (*η*) of approximately 4.07% is obtained. The *η* improvement can be attributed primarily to the morphology effect of ZnO nanocones on light-trapping and effectively passivating the interface surface recombination sites of ZnO nanocones by coating with a ZnS shell layer.

## Introduction

Zinc oxide nanostructures have attracted significant attention because of their desirable properties for various device applications [1–4]. ZnO nanomaterials show several advantages such as simple tailoring of the structures, high carrier mobility, ease of crystallization, and facile, as well as low-cost, large-scale production [5–7].

ZnO nanorod arrays, as antireflectors synthesized on Si wafers, could achieve a reflectance of less than 10% in broad band reflection suppression from 400 nm–1200 nm for a tapered nanorod array with average tip diameter of 10 nm. Effective antireflection coatings can enhance the conversion efficiency of Si solar cell through increased light coupling [8,9]. In the present study, a novel type of ZnO nanocone structures that can significantly reduce light reflection from the UV to near-infrared region is introduced. The ZnO nanocones with superior antireflection structure have great potential in light harvesting and electro-optical device applications [10].

Various 1-D nanostructure materials have been utilized as potential photoanode materials for of dye-sensitized solar cells (DSSCs) [11,12]. Among these materials, ZnO has been an ideal alternative to TiO2 because the electron mobility of ZnO has been proven to be higher than that of TiO_2_, which means lower charge recombination [13–15]. Furthermore, the well-aligned ZnO nanostructures can be simply synthesized and its morphology can be modified [16]. Some of techniques are easily scalable and appropriate for many substrates [17], which makes ZnO very attractive for fabricating DSSCs using low-cost solution methods. Although the conversion efficiency of the ZnO-based DSSCs is still lower than that of TiO_2_-based DSSCs, much effort has been focused to improve the performance of ZnO-based DSSCs [18–20].

Introducing a protective layer on the ZnO and simultaneous increasing the surface area by surface treatment should be a promising approach to improve the performance of DSSCs [21]. Numerous studies have focused on the type II band alignment of heterostructures because these structures can induce charge separation at the interface of two different materials and localize the electron and hole into different spatial regions of the nanostructure, thereby increasing the carrier lifetimes [22,23]. Cell efficiency can be changed depending on the type of shell material. ZnO nanowires coated with Al_2_O_3_ reduce power conversion efficiency because of the insulating barrier of Al_2_O_3_ shell layer that decreases injection efficiency [24]. Coating a semiconductor shell with a wide band gap has been proposed to be an effective method for suppressing surface recombination by passivating the surface defects. A Zn-based material may be a suitable shell layer on ZnO nanowires because it can help prevent the shell layer from acting as an insulating layer and causing a decrease in short-circuit current (*J*_SC_).

ZnO-based core–shell nanostructures, including ZnO–ZnSe, ZnO–ZnTe, ZnO–TiO_2_, and ZnO–ZnS have been investigated [25–28]. However, enhancing the overall efficiency of DSSCs remains a challenge. Traditional preparation techniques are disadvantageous because of special equipment or high temperature requirements, resulting in a high-cost and energy consuming synthesis process. Thus, simple, low-cost, and low-temperature techniques are necessary. In this study, cone-shaped ZnO–ZnS core–shell heterostructures were successfully fabricated using a facile two-step approach that combined electric field-assisted aqueous solution (EFAS) process and aqueous solution method. Moreover, the broad-band antireflective properties of self-assembled vertically aligned ZnO and ZnS-coated ZnO nanocones grown on fluorine-doped tin oxide (FTO) substrates are reported. DSSCs based on ZnO–ZnS core–shell nanocone arrays were assembled, and the effects of morphology and shelled structures on the efficiency of the DSSCs were investigated.

## Materials and Methods

First, vertically aligned ZnO nanorod and nanocone arrays were grown on FTO substrates using the EFAS method [29]. A radiofrequency magnetron sputtering system was used to deposit a ZnO seed layer onto the films. Sputtering was performed at an incident power of 100 W for 1 h at 100°C using a ceramic ZnO target with 99.99% purity. A two-electrode setup was used in the electrochemical deposition of the ZnO nanostructures on the substrates. Zinc nitrate hexahydrate [Zn(NO_3_)_2_ 6H_2_O] and hexamethylenetetramine (C_6_H_12_N_4_) were individually dissolved in deionized (DI) water at equal molar concentrations at room temperature. After mixing and uniform stirring, the aqueous solution (10 mM) was transferred into an electrochemical cell. The seed layer-coated ZnO/FTO film and platinum electrode were immersed into the aqueous solution as the cathode and anode, respectively.

Growth time and temperature were maintained at 40 min and 110°C, respectively. The FTO substrates containing the ZnO nanorods were then rinsed thoroughly with DI water and annealed at 250°C in air for 1 h to remove any organic molecules present on the surface of the nanorods. The as-grown ZnO nanocone arrays were then used as a template in the second growth process. Subsequently, 0.2 M thioacetamide aqueous solution was used as the sulfur source. The resulting solution was transferred into a beaker and then sulfidization was performed at 80°C growth temperature for 3 h in a conventional laboratory oven. After the growth process, the final products were repeatedly rinsed with DI water to remove possible impurities and then dried at room temperature.

Dye sensitization was performed by immersing the ZnO and ZnO–ZnS core–shell electrodes in the dye bath containing C218 (0.1 mM) dye in a solvent mixture of acetonitrile and butanol (1:1, v/v) for 2 h. Co-adsorbent 3α-7α-dihydroxy-5β-cholic acid (1 mM) was added in the C218 dye solution to reduce the dye aggregation on the ZnO and ZnO–ZnS core–shell films. The sensitized ZnO and ZnO–ZnS nanostructure electrodes were assembled with a platinum-coated FTO counter electrode, and the gap between the two electrodes was controlled by a 25 μm thick Surlyn film. A drop of electrolyte solution containing 0.06 M I_2_, 0.2 M NaI, 0.6 M 1-propyl-3-methylimidazolium iodide, and 0.5 M 4-*tert*-butylpyridine in 3-methoxypropionitrile was introduced into the gap between the two electrodes. The active area of all the DSSCs was 0.25 cm^2^.

The samples were characterized by field-emission scanning electron microscopy (FESEM, FEI Sirion 200) and transmission electron microscopy (TEM, Philips CM12, FEI, CO). X-ray diffraction (XRD, Rigaku Ultima IV) was employed to determine the crystallinity of the ZnO nanostructures. The transmittance property was characterized using a UV–vis spectrophotometer (Perkin Elmer Lambda 750). The photovoltaic characteristics of the DSSC devices were measured using a Solar Simulator (150 W simulator, Bunkoh-Keiki Co. Ltd.) under simulated solar light with a xenon lamp power supply (AM 1.5, 100 mW/cm^2^). The dye absorbed amounts on ZnO-based photoelectrodes were determined by measuring the UV–vis absorption spectra of solutions containing dyes desorbed from the samples. The charge transfer and recombination processes were also investigated by electrochemical impedance spectroscopy (EIS) at frequencies ranging from 0.1 Hz to 0.1 MHz with oscillation potential amplitudes of 10 mV.

## Results and Discussion

**Fig 1:**
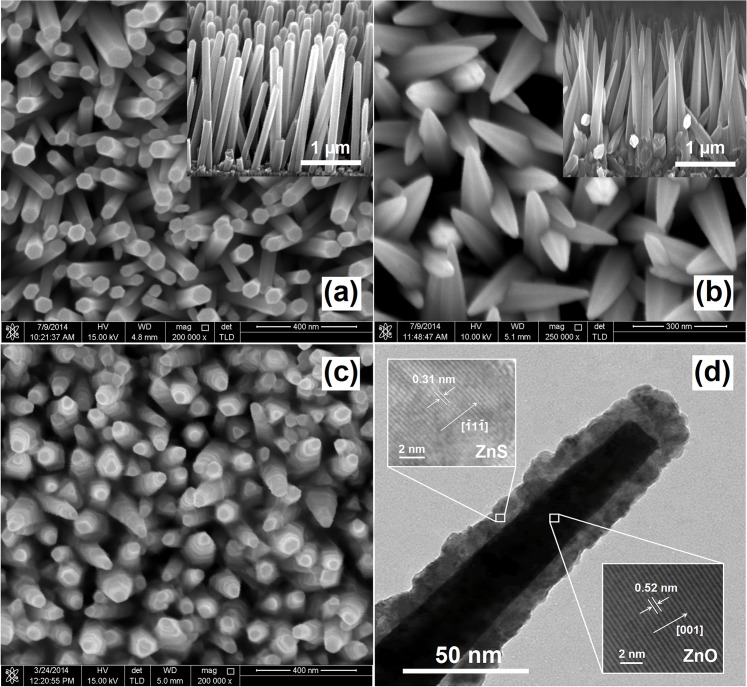
FESEM and HRTEM images of ZnO-based nanostructures. FESEM images of (a) ZnO nanorods, (b) ZnO nanocons, (c) ZnO–ZnS core–shell nanocones, and (d) HRTEM image of the ZnO-ZnS core-shell structure.

Fig 1 and 1B show the FESEM micrographs of the as-grown ZnO nanorod and nanocone arrays, respectively. Cone-shaped nanorods can be obtained by efficiently controlling the current density in EFAS process which is fully explained in our previous research [29]. At low current densities, the hydrothermal growth treatment of ZnO facilitated the growth of hexagonal-shaped nanorod arrays on the substrate. When current densities gradually increased, the growth rate of (0002) plane was much higher than that of non-polar surfaces. The fastest growing planes easily disappeared and finally resulted in the crystal shape build up only by slow growth rate facets. Consequently, the ZnO nanorods became smaller in diameter at the tip and had tapered ends.

Fig 1B clearly shows that the ZnO nanocones with sharp tips grew vertically on the FTO substrate. Further analysis indicates that the average length of the nanoneedles was approximately 2 μm, and the diameters were 15 and 150 nm at the tip and base, respectively. The FESEM micrograph of the nanocone arrays after the sulfidization of ZnO is presented in Fig 1C. The ZnS layer with a thickness of several tens of nanometers was distributed uniformly over the entire nanocone. Fig 1D illustrates a high-magnification TEM micrograph of a ZnO–ZnS core–shell nanocone, indicating that the ZnS shell was deposited directly in the radial direction from the surface of the ZnO core. From the enlarged image inserted in Fig 1D, the interplanar spacing of 0.31 nm corresponds to the [111] lattice plane of ZnS. Furthermore, the lattice spacing of 0.52 nm corresponds to the *d*-spacing of (0002) crystal planes, which confirms that the ZnO–ZnS core–shell nanocones are mainly oriented in the c-axis direction.

**Fig 2:**
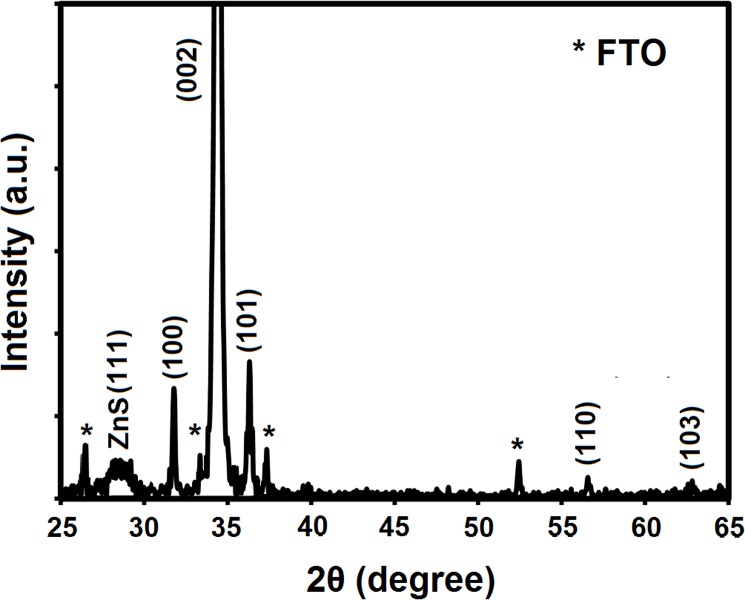
XRD patterns of the ZnO–ZnS core-shell nanocone arrays on FTO substrate.

Fig 2 shows the XRD pattern of the ZnO–ZnS core–shell nanocone arrays. The diffraction peaks were identified and matched with the hexagonal wurtzite crystal structure (ICSD 01-080-0074). The predominant (0002) peak with narrow width indicates well oriented along the normal direction of the substrate surface, which is consistent with the FESEM observation in Fig 1. An additional relatively broad and weak peak was observed at 2θ = 28.6°, which corresponds to the ZnS phase. The average nanocrystallite size of ZnS products is estimated to be about 2.7 nm by employing the well-known Debye–Scherrer formula which is close to excitonic Bohr radius of ZnS which is about 2.5 nm [30].

**Fig 3:**
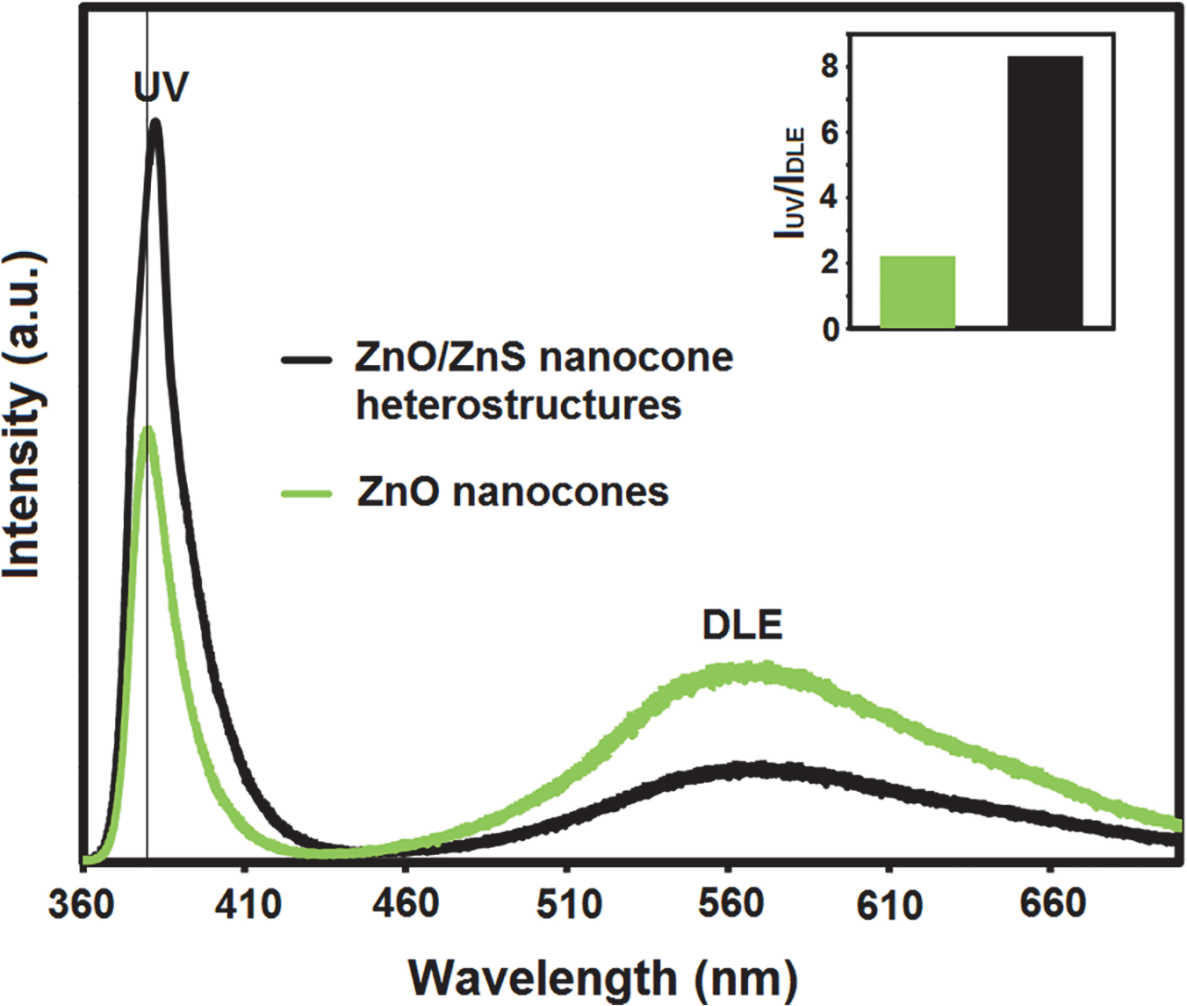
Room-temperature PL spectra of the bare ZnO and the shelled ZnO nanocones. The inset shows the relative PL intensity ratio between the peak intensity of UV and DLE.

The photoluminescence (PL) was characterized at room temperature to investigate the optical properties of ZnO–ZnS core–shell nanocone arrays. Fig 3 shows the PL spectra of the shelled and bare ZnO nanocones (as the reference). Two emitting bands were observed in the ZnO nanocone arrays, including a narrow UV emission peak at 380 nm from the excitonic recombination associated with the near-band edge emission of ZnO [31,32] and a broad green emission centered around 580 nm that could be attributed to the Zn-vacancy, O-vacancy, and extrinsic impurities [33].

**Fig 4:**
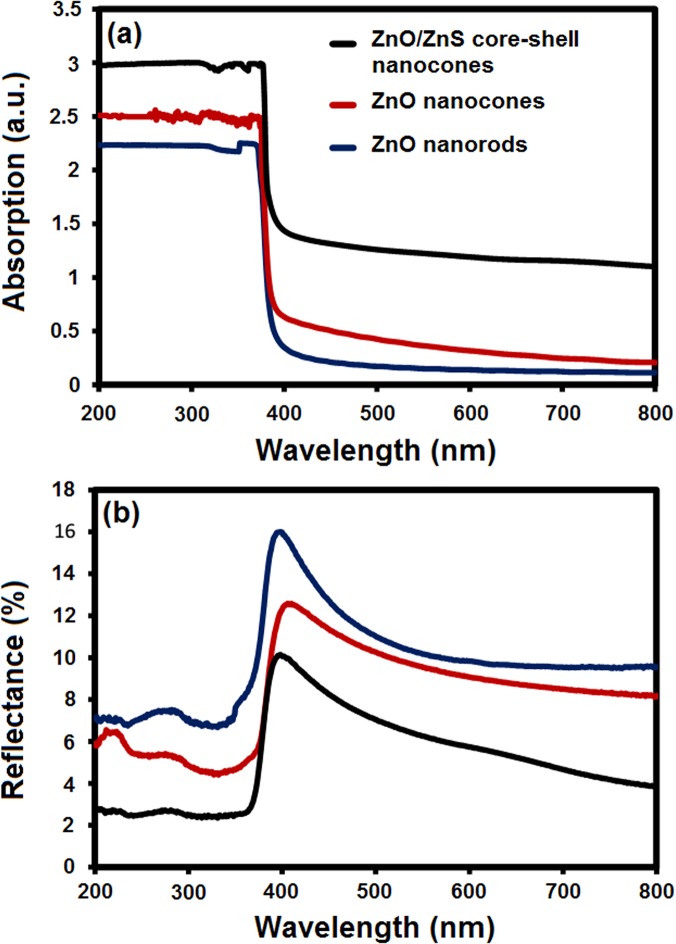
The band-edge alignments at the heterointerface between ZnO and ZnS semiconductors.

The ZnO–ZnS core–shell nanocones underwent a significant enhancement in the luminescence intensity and a small redshift in the UV region (Fig 3). The redshift in the PL spectrum may be ascribed to the strain caused by the lattice mismatch between ZnO and ZnS. Theoretically, the strain in the ZnO–ZnS interface is strong enough to reduce the total band gap of the system [34] (Fig 4). Furthermore, a thin layer of a wider band gap semiconductor on the ZnO nanocones surface passivated the surface electronic states of the cores, thereby enhancing the PL intensity of the UV emission region [35].

In addition, the redshift of the free exciton emission confirmed the presence of S atoms that were diffused into the ZnO surface to shape the isoelectronic centers. The S atoms filled up the oxygen vacancies on the ZnO nanocone surface, thereby resulting in the decreased number of oxygen vacancies and improved luminescence efficiency [36].

The intensity ratios of the green emission to the UV emission of the bare ZnO and shelled ZnO nanocones are shown in the inset of Fig 3. The ratio of green emission was obviously reduced in the ZnO–ZnS core–shell nanostructure. The reduced emission could be attributed to the reduction in oxygen vacancies in the ZnO core. Given that oxygen ions are formed during sulfidization and external diffusion of oxygen ions is difficult because of the large ionic radius, a considerable number of oxygen ions could be captured within the intermediate gap. Therefore, oxygen ions could fill the oxygen vacancies in the ZnO core, thereby decreasing the number of oxygen vacancies.

The results show that the UV emission intensity was significantly enhanced in the shelled ZnO nanocones. Therefore, the shelled ZnO nanocones are more applicable for fabrication of optoelectronic devices, such as UV light-emitting diodes and lasers.

**Fig 5:**
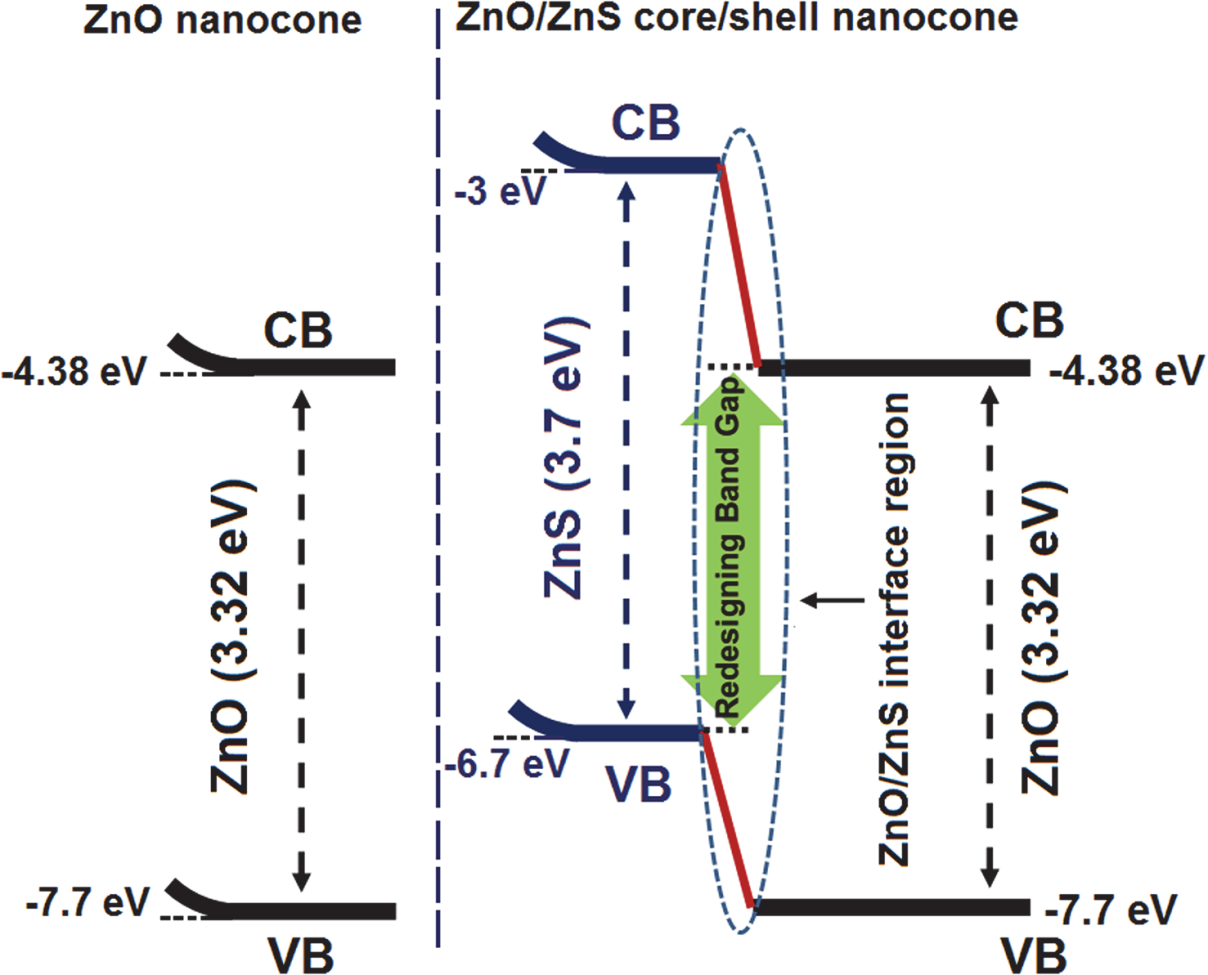
Optical absorption and reflectance spectra of the samples. (a) Absorption and (b) reflectance spectra of the ZnO nanorods, nanocons, and ZnO–ZnS core–shell nanocones.

The optical absorption and reflectance spectra of the samples are shown in Fig 5. The bare ZnO nanocones exhibited remarkably enhanced absorption compared with the nanorod arrays (Fig 5A), which are also significantly comparable with previous reports on ZnO nanowires and films [37]. These results indicate the antireflective and the absorption enhancement that can be attributed to the gradually decreasing diameter of the ZnO nanocones from the root to the top, which led to the graded transition of the effective refractive index [38].

Furthermore, the results may be associated with the light scattering into large angles beyond the angle of total reflection [39]. These findings suggest that the well-aligned ZnO nanocone arrays display a strong light trapping effect, which is advantageous for photovoltaic cell applications.

The ZnO–ZnS core–shell nanocones show higher absorption than the bare ZnO nanocones and nanorods (Fig 5A). The energy band structure of ZnO and ZnO–ZnS core–shell nanocones can be schematically illustrated in Fig 4. The optical absorption increased after the ZnS shell formation because of the lower band gap of the ZnO–ZnS interface compared with either semiconductor. The charge interchange produced a lattice strain in the interface and exhibited much lower band gap than the actual band gap of ZnO and ZnS. The crystal structure relaxation produced delocalization in the bottom of the conduction band and resulted in a narrow band gap [40].

Furthermore, the formation of ZnS bonds in the ZnO lattice strongly affected the bowing in valence band edge and reduced the overall band gap of the system. The FESEM and HRTEM micrograph shows that the surface of the core–shell nanocones became rough because of the newly formed ZnS shell, which decreased the reflection of the incident light. This phenomenon indicates that ZnS coating on ZnO nanocones with high surface roughness should be an ideal photoanode structure for the ZnO–ZnS core–shell-based DSSCs.

**Fig 6:**
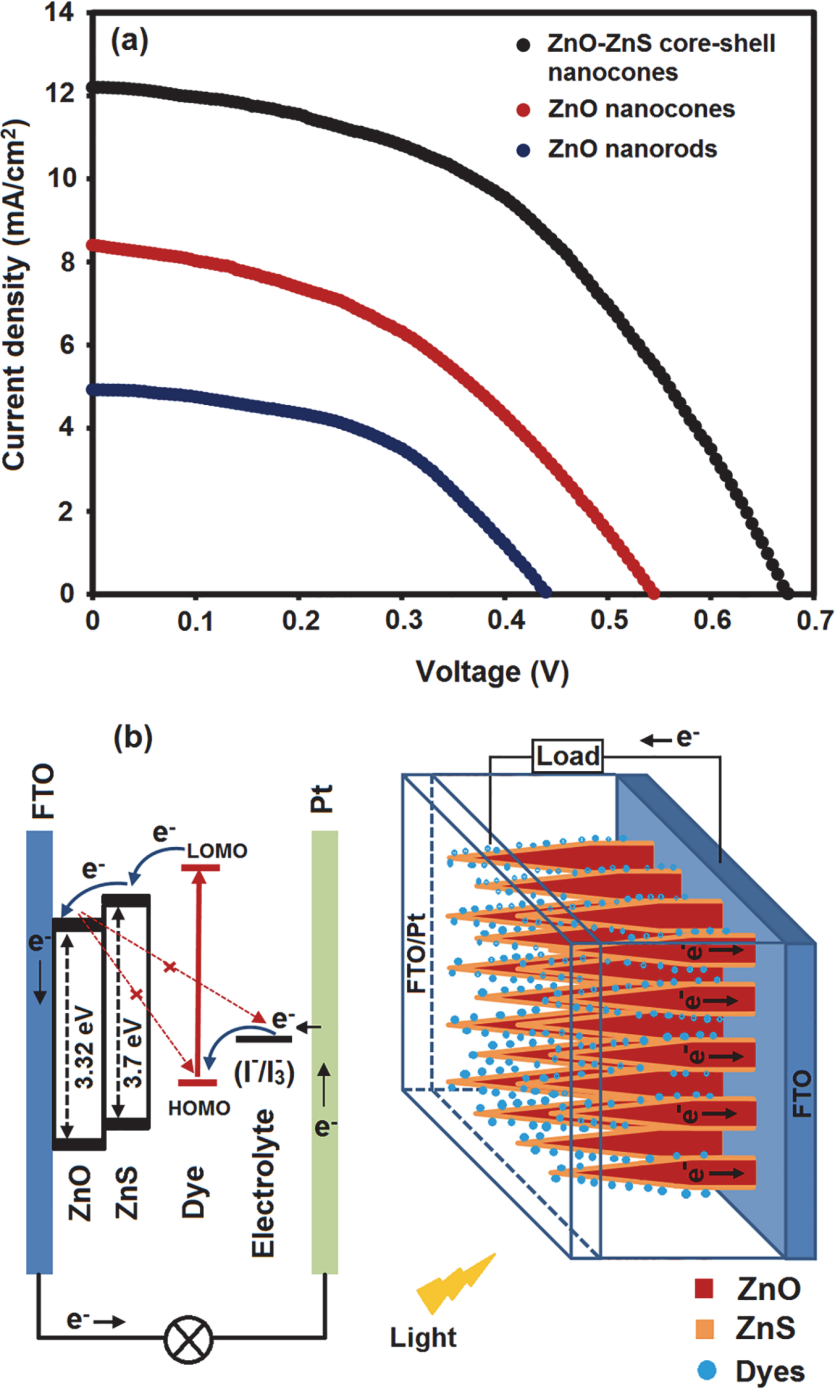
The photocurrent density–voltage characteristics of the ZnO nanostructures (a) Comparison of photocurrent density-voltage plot of the DSSCs based on ZnO nanorods, nanocons, and ZnO–ZnS nanocones (b) schematic diagram of the energy levels of the hetrostructure ZnO/ZnS photoelectrode in a DSSC.

The photovoltaic properties of the ZnO nanorods, nanocones, and core–shell structures were investigated by assembling the DSSCs and performing the standard methods described in the experimental section. The photocurrent density–voltage characteristics of the ZnO nanostructures are shown in Fig 6. The related physical values, such as short-circuit current (*J*_*SC*_), open-circuit voltage (*V*_*OC*_), fill factor (*FF*), and overall light to electrical energy conversion efficiency (*η*), are summarized in Table 1. The *η* value can be calculated by the following equation [41]:

$$\eta =\;\left(FF\times J_{SC}\times V_{OC}\right)/P_{in}$$ 1

From Table 1, the ZnO nanocones cell exhibited the highest efficiency (*η* = 2.09%), which was almost twice that of the ZnO nanorods cell (*η* = 0.93%). This characteristic could be attributed to the higher *V*_*OC*_ and *J*_*SC*_. The *J*_*SC*_ of a DSSC depends on various factors, which include the electron injection efficiency, the light harvesting efficiency, and the electron collection efficiency [42]. Given that ZnO nanocones had lower surface areas with lower dye loading than ZnO nanorods (1.93×10^–8^ mol/cm^2^), and the ZnO nanocones dimensions were comparable to those of the ZnO nanorods [43]. Thus, a lower light harvesting efficiency is expected for the ZnO nanocones cells compared with the ZnO nanorods cells. Consequently, higher *J*_SC_ of the ZnO nanocone-based cells was obtained because of the predominance of the exposed reactive {101̅1} facets, which improved the electron collection process and electron injection in the device [44].

**Table 1:** Performances of the DSSCs based on ZnO nanorods, nanocons, and heterostructure ZnO–ZnS nanocones.

Cells	Jsc (mA/cm^2^)	Voc (V)	FF	η (%)	dye loading (10^−8^ mol/cm^2^)
ZnO nanorods	4.9	0.43	0.44	0.93	1.93
ZnO nanocones	8.4	0.54	0.46	2.09	1.58
ZnO-ZnS core-shell nanocones	12.2	0.68	0.49	4.07	3.67

An energy conversion efficiency of approximately 4.07%, which was much higher than those of the ZnO nanocone- and nanorod-based DSSCs, was obtained in the ZnO–ZnS core–shell nanocone-based DSSCs. The significant performance enhancement in the ZnO–ZnS-based DSSCs could be attributed to the core–shell structure, which consists of nanocones and nanograins. This characteristic resulted in the enhanced an internal surface area for the dye-loading (3.67×10^–8^ mol/cm^2^; almost two times higher than that of both the ZnO nanorod and ZnO nanocone photoanodes) and a remarkable increase in *J*_SC_.

However, the S atoms in the ZnS layer filled up the oxygen vacancies in the ZnO nanocone [45], which led to the reduction in the recombination of electrons in the DSSCs. Given that the high band gap of the ZnS shell material suppressed the tunneling of the electrons from the ZnO core material to the ZnS shell layer, excited electrons were completely confined inside the ZnO. Moreover, the ZnS layer on the ZnO nanocone formed a type-II band alignment. The interface of the ZnO–ZnS heterostructure led to the large elastic strain, which might have increased the oscillator strength; in addition, the individual band gap could be smaller than the initial value (Fig 4) [46]. According to the thermodynamic theory, the variation in the free energy for the electron transfer from the excited dye molecules (LOMO) to the conduction band of ZnO–ZnS heterostructure is more positive than that of bare ZnO nanocones. However, the changes in the free energy for the recombination of coated ZnO nanocones were less positive than that of bare ZnO. Consequently, dark exciton formation in the ZnO–ZnS nanocone arrays reduced the recombination rate of excitons such that the ZnS layer improved the carrier collection and significantly increased the current density in the photoanode materials [34].

**Fig 7:**
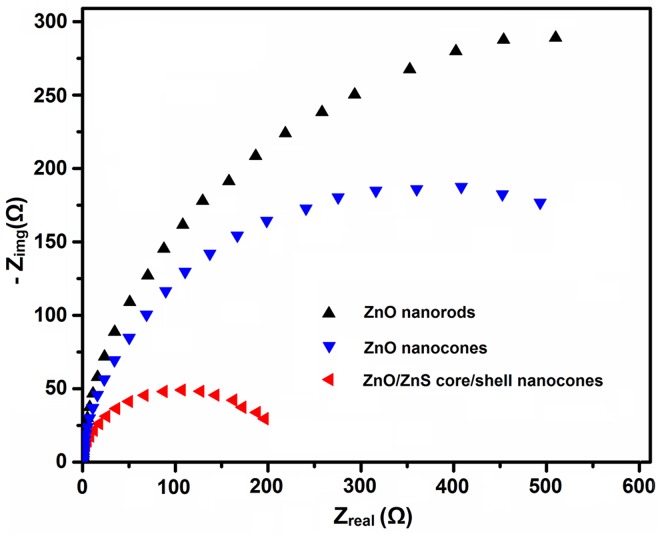
EIS Nyquist plots of DSSCs based on different ZnO nanostructures.

It is well known that EIS is a powerful tool to study charge separation/transfer and recombination processes in solar cells. EIS characterizations were carried out to further clarify the shell effect on photogenerated charge separation processes of ZnO nanocone arrays. The radius of the arc on the EIS spectra represents the electron transfer resistance at the surface of electrodes. In Fig 7, the arc radius on EIS Nyquist plot of the ZnO/ZnS core/shell nanowires is smaller than that of the ZnO nanorods and cones, which suggest that ZnS shell leads to a more effective charge separation and a faster interfacial charge transfer. These EIS results clearly indicate that the introduction of ZnS shell to ZnO nanocones can effectively improve the photogenerated charge separation process.

## Conclusions

A new type of high-antireflection ZnO–ZnS heterostructure arrays was synthesized on an FTO substrate using a facile and cost-effective two-step approach that combined EFAS and aqueous solution methods. The optical absorption and reflectance spectra of the samples indicated that the antireflective property of the ZnO–ZnS core–shell nanocone was markedly higher than that of any previously obtained ZnO structures. The PL spectrum demonstrated a considerable enhancement in the luminescence intensity and a small redshift in the UV region. This phenomenon could be attributed to the strain caused by the lattice mismatch between ZnO and ZnS. The DSSCs assembled with C218 dye-coated ZnO–ZnS core–shell nanocone photoanodes exhibited the highest energy conversion efficiency of about 4.07%. The improved efficiency could be explained by the enhancement of light-trapping effect caused by the cone-shaped ZnO nanostructures and effective retardation of the charge recombination process by the ZnS coating on the ZnO nanocones. These results indicate that vertically aligned ZnO–ZnS core–shell nanocone arrays can be potentially applied to optoelectronic devices, such as solar cells, because of their higher surface-to-volume ratio, better light-trapping effect, and longer carrier lifetime than any other structures.
